# The Human Adenovirus 5 L4 Promoter Is Activated by Cellular Stress Response Protein p53

**DOI:** 10.1128/JVI.01924-13

**Published:** 2013-11

**Authors:** Jordan Wright, Keith N. Leppard

**Affiliations:** School of Life Sciences, University of Warwick, Coventry, United Kingdom

## Abstract

During adenovirus infection, the emphasis of gene expression switches from early genes to late genes in a highly regulated manner. Two gene products, L4-22K and L4-33K, contribute to this switch by activating the major late transcription unit (MLTU) and regulating the splicing of its transcript. L4-22K and L4-33K expression is driven initially by a recently described L4 promoter (L4P) embedded within the MLTU that is activated by early and intermediate viral factors: E1A, E4 Orf3, and IVa2. Here we show that this promoter is also significantly activated by the cellular stress response regulator, p53. Exogenous expression of p53 activated L4P in reporter assays, while depletion of endogenous p53 inhibited the induction of L4P by viral activators. Chromatin immunoprecipitation studies showed that p53 associates with L4P and that during adenovirus type 5 (Ad5) infection, this association peaks at 12 h postinfection, coinciding with the phase of the infectious cycle when L4P is active, and is then lost as MLP activation commences. p53 activation of L4P is significant during Ad5 infection, since depletion of p53 prior to infection of either immortalized or normal cells led to severely reduced late gene expression. The association of p53 with L4P is transient due to the action of products of L4P activity (L4-22K/33K), which establish a negative feedback loop that ensures the transient activity of L4P at the start of the late phase and contributes to an efficient switch from early- to late-phase virus gene expression.

## INTRODUCTION

Adenovirus infection proceeds through a coordinated pattern of gene expression in order to generate the intracellular conditions necessary for successful virus replication. Gene expression is broadly separated into early and late phases, each distinguished by the onset of expression of specific genes. Whereas early-phase gene products are primarily concerned with providing the ideal environment for viral DNA replication, the late genes encode predominantly the structural proteins that allow the assembly of the virus particle (1). Gene expression switches from early to late via a small class of intermediate genes that become activated around the time that viral DNA replication begins (2–4). These events have been most widely studied in human adenovirus type 5 (HAdV-C5 [Ad5]). However, though many factors contributing to this early-late switch are known, a full understanding of the mechanism remains elusive.

The majority of Ad5 late proteins are encoded within the major late transcription unit (MLTU), which is driven by the major late promoter (MLP) (5, 6). Within the MLTU, five groups of mRNA, termed L1 to L5, are defined by distinct poly(A) sites. MLP activation is achieved upon the onset of viral DNA replication by the intermediate gene products IX and IVa2, the latter in conjunction with the L4 products 22K and/or 33K (7–9). IVa2 expression commences after an unknown cellular repressor bound to its promoter is titrated out by the excess of nascent viral genomes (2, 3). Upon activation of the MLP, L4-22K and L4-33K additionally contribute to the correct expression of the full repertoire of adenovirus late proteins by influencing the splicing of the MLTU pre-mRNA (10–12); L4-22K also cooperates with IVa2 to promote packaging of viral DNA into nascent capsids (7). Both L4-22K- and L4-33K-deficient viruses display defects in late gene expression and efficiency of packaging (13–15), emphasizing the importance of these proteins for efficient replication at multiple levels.

The paradoxical requirement of two MLTU products, L4-22K and L4-33K, for MLP activity was resolved by the discovery of a novel promoter (L4P) embedded within the L4-100K open reading frame that was sufficient to drive expression of both L4-22K and L4-33K (16). Deletion of the L4P results in severely reduced virus late gene expression due to the loss of L4-22K and L4-33K functions. L4P is stimulated upon exogenous expression of E1A, Orf3, and IVa2 (16). However, this is unlikely to be the entire repertoire of regulatory proteins required for full L4P activity, as transfection of cells by fragmented DNA of both viral and nonviral origin also stimulates L4P (16).

The cellular protein p53 is a transcription factor that is considered to be a global regulator of cellular responses to stress and is consequently a tumor suppressor. p53 accumulation and activation by posttranslational modifications are induced by insults to the cell, including those that result in DNA damage. Once activated, p53 causes cell cycle arrest and, depending on the extent of damage, apoptosis. These effects are mediated by the direct binding of activated p53 to the promoters of its target genes, where it can act as either a transcriptional activator or repressor (reviewed in reference 17). Ad infection activates p53, and the virus must therefore overcome its proapoptotic effects by inhibiting p53 transcriptional activity in order to successfully replicate (18, 19). Early studies indicated that the Ad5 E1B-55K was the protein responsible for achieving this, as it selectively binds p53 and is able to inhibit its transactivating ability as well as to promote p53 export to the cytoplasm (20–22). However, further analyses have shown that E1B-55K is dispensable for adenovirus-mediated inhibition of the p53 transcriptional program, indicating a role of other virus proteins, such as E4 Orf3 (23–25). At later times in infection, E1B-55K acts in conjunction with E4 Orf6 and cellular cullin 5, among other cellular proteins, to degrade p53 and other cellular targets in a proteasome-dependent manner (26–28).

Despite the evidence that p53 activity would be deleterious to the outcome of infection, some studies have indicated that p53 may play a positive role in the life cycle of adenovirus by enhancing late gene expression and increasing the cytopathic effect (29–31). Conversely, a recent study using two primary cell types suggested that p53 depletion did not affect the production of viral progeny (32). It is therefore likely that the relationship between adenovirus infection and p53 is more complex than is currently understood.

Intriguingly, although the overall thrust of Ad5 functions is to block p53 activity, it has been reported that there is a brief period prior to E4 Orf6 expression when the E1B-55K-mediated repression of p53 is relieved by the viral E4 Orf3 protein (33). However, its effects on cellular genes is still inhibited, as E4 Orf3 induces histone H3K9 trimethylation specifically at p53-responsive promoters (25). This leads to the formation of heterochromatin at the promoters and the consequent downregulation of the respective genes. Thus, although p53 is nuclear and potentially transcriptionally active at this stage in Ad5 infection, it is unable to exert its effects on endogenous promoters.

In this study, we demonstrate that p53 is a significant activator of L4P, and hence of adenovirus late gene expression, during the time window in which its repression by E1B-55K is transiently relieved. Overexpression of p53 increased L4P activity, whereas endogenous p53 depletion reduced its activity. Furthermore, endogenous p53 associated with the L4P for a brief period during infection that correlated with the onset of late gene expression, and depletion of p53 severely reduced entry into the late phase of virus gene expression. Finally, the two products of L4P activation, L4-22K and L4-33K, inhibit both p53 association with the L4P and also its ability to activate this promoter, indicating the presence of a negative feedback mechanism controlling L4P.

## MATERIALS AND METHODS

### Viruses and plasmids.

All infections were carried out using wild-type adenovirus type 5 strain wt300 (34) at a multiplicity of 1 or 10 fluorescence foci units (FFU) per cell. The Ad5 genomic clones pTG3602-Ad5wt (35) and pTG3602-Ad5ΔL4P (16) were described previously. Expression plasmids pMEPCMV-IVa2 (36), pCMV22KFLAG, pCMV33KFLAG (12), pcDNA3.1Orf3 (37), and pcDNA-p53 (38) were described previously. pcDNAHisLacZ was obtained from Invitrogen. L4P luciferase reporter plasmids were constructed using pGL3-Basic vector (Promega) as previously described (16). pCI-Neo (empty vector) was obtained from Promega.

### siRNA and antibodies.

All small interfering RNAs (siRNAs) were obtained from Ambion. p53 knockdown was achieved using a validated p53 siRNA (s605) (sense strand; 5′-GUAAUCUACUGGGACGGAATT-3′). The control siRNA was designed to have no sequence similarity to any Homo sapiens or Ad5 sequence using Ambion siRNA target finder (sense strand; 5′-GAGCCGGACGGCCAAAGAAAUU-3′).

Proteins were detected by Western blotting using the following antibodies: 1:10,000 mouse anti-p53 (DO-1; Santa Cruz), 1:10,000 AdJLB (Ad late proteins) (11), 1:10,000 rabbit anti-β-actin (13E5; Cell Signaling), and 1:5,000 mouse anti-E1A (M73) (39). Secondary horseradish peroxidase (HRP)-conjugated antibodies were 1:10,000 goat anti-mouse IgG (Sigma) and 1:50,000 goat anti-rabbit IgG (Santa Cruz). For chromatin immunoprecipitation, the antibodies used were mouse anti-p53 (DO-1; Santa Cruz) and an IgG2A isotype control (control IgG, MAB003; R&D Systems).

### Cell culture and transfection.

293 and HeLa cells were maintained at 37°C and 5% CO_2_ in Dulbecco's modified Eagle medium (DMEM) supplemented with 10% newborn bovine serum. U2OS cells were maintained in McCoy's 5A medium supplemented with 10% fetal bovine serum. MRC5 cells (40) were maintained in modified Eagle medium supplemented with 10% fetal bovine serum, 1% nonessential amino acids, and 2 mM l-glutamine. All cells were seeded 24 h prior to the beginning of the respective procedure. Plasmid and virus DNA transfections were carried out in 6-, 12-, and 24-well plates using Transit-LT1 (Mirus) according to the manufacturer's instructions at a ratio of 3 μl of lipid reagent per μg of DNA. siRNA transfections were carried out using Lipofectamine 2000 according to the manufacturer's instructions at a ratio of 2 μl of lipid reagent per μl of siRNA (at 50 μM) used. Transfections within an experiment were normalized to a standard amount of input DNA by addition of empty vector. All siRNA transfections were performed with 25 to 50 pmol siRNA (50 nM final concentration).

### Luciferase assays.

Cells were harvested for reporter analyses with 1× reporter lysis buffer (Promega) according to the manufacturer's instructions. For each sample, relative luciferase activity (RLA) was calculated by normalizing the luciferase activity to β-galactosidase activity, which served as a control for transfection efficiency. Within an experiment, all RLA values were then expressed as fold activity relative to the respective control. In the figures, error bars indicate the standard deviations for three replicates within an experiment. All experiments are representative of multiple independent repeat experiments.

### SDS-PAGE and Western blotting.

Whole-cell lysates were harvested in 1× SDS-PAGE sample buffer and typically 5 to 10% of the total volume was separated in 10% acrylamide gels by SDS-PAGE. Proteins were transferred to Hybond-ECL nitrocellulose membranes (GE Healthcare). Western blot analysis was carried out as previously described using HRP-conjugated secondary antibodies and SuperSignal West Femto maximum sensitivity chemiluminescence reagent (Thermo Scientific) (41).

### Chromatin immunoprecipitation (ChIP) and quantitative PCR.

Cells (1 × 10^7^) were fixed for ChIP by incubation with 1% formaldehyde in culture medium for 10 min. The formaldehyde was then quenched by incubation with 150 mM glycine for 5 min. Cells were then washed twice in ice-cold phosphate-buffered saline (PBS) containing 1 μg/ml aprotinin and collected by low-speed centrifugation. Cell pellets were resuspended in 1 ml ice-cold buffer C (20 mM HEPES [pH 7.9], 420 mM NaCl, 1.5 mM MgCl_2_, 0.2 mM EDTA, 25% glycerol) containing 1 μg/ml aprotinin and left on ice for 20 min with occasional mixing. Nuclei were then pelleted by centrifugation at 16,000 × *g* at 4°C for 10 min before resuspension in 120 μl breaking buffer (50 mM Tris-HCl [pH 8], 150 mM NaCl, 1% SDS, 2% Triton X-100, 1 mM EDTA) and then left on ice for 10 min. These suspensions of nuclei were then sonicated on ice with six 40-s pulsed bursts (microtip, 25% output energy; Jencons Ultrasonic Processor). Sonicated samples were diluted 10-fold with Triton Buffer (50 mM Tris-HCl [pH 8], 150 mM NaCl, 1 mM EDTA, 0.1% Triton X-100), and cellular debris was pelleted by centrifugation at 16,000 × *g* for 10 min. The supernatants were then precleared with protein A Dynabeads (Invitrogen; prepared by preincubation with salmon sperm DNA in Triton buffer containing 1% bovine serum albumin [BSA]) for 2 h prior to incubation in equal aliquots overnight with 1 μg test or control antibodies. Antibody-protein-DNA conjugates were precipitated by incubation with Dynabeads for 1 h and washed five times with ChIP wash buffer (50 mM Tris-HCl [pH 8], 750 mM NaCl, 5 mM EDTA, 1% Triton X-100, 0.1% sodium deoxycholate, 0.1% SDS), twice with LiCl buffer (10 mM Tris-HCl [pH 8], 0.25 M LiCl, 1 mM EDTA, 0.5% Triton X-100, 1% sodium deoxycholate, 1% SDS), and twice with TE buffer (10 mM Tris-HCl [pH 8], 1 mM EDTA). Washed beads were then incubated at 65°C for 4 h in SDS-NaCl-DTT buffer (62.5 mM Tris-HCl [pH 6.8], 200 mM NaCl, 2% SDS, 10 mM dithiothreitol) and then for 1 h at 50°C with the addition of 1 μg proteinase K. The immunoprecipitated DNA was then purified by two rounds of phenol-chloroform extraction and ethanol precipitation before being resuspended in water.

Quantitative PCR was carried out on an ABI SDS 7000 system using standard SYBR green cycling conditions and associated software. Reactions were performed using SYBR green PCR Mastermix (Applied Biosystems) or SYBR green JumpStart *Taq* ReadyMix (Sigma-Aldrich) according to the manufacturers' instructions. All primer sets were validated by standard curve analysis to calculate amplification efficiency prior to assay: Ad25887F, 5′-GTTGAAACTCACTCCGGGGCTGT-3′; Ad26125R, 5′-CGCCGGACTGGGGGTCCAA-3′; Ad26018R, 5′-CGCAGGCGGTAAGCTCCGCAT-3′; Ad26018F, 5′-CATTACCCAGGGCCACATTC-3′; Ad26098R, 5′-CCCGTCCCTTTCGTAGCA-3′. For analyses of ChIP following plasmid transfections, the amount of immunoprecipitated DNA from test or control antibodies was first expressed as a percentage of input by using the differences in *C_T_* (cycle threshold) values of immunoprecipitated and input reactions and the known efficiency of the respective primer set. For each sample, the percentage of DNA pulldown by the test antibody was then corrected for background by subtracting the value obtained from the control antibody. For ChIP from infected cells, for each sample the amount of DNA immunoprecipitated by the test antibody was expressed relative to the amount of DNA immunoprecipitated by the control antibody (fold enrichment).

## RESULTS

### The L4 promoter is activated by exogenously and endogenously expressed p53.

**Fig 1:**
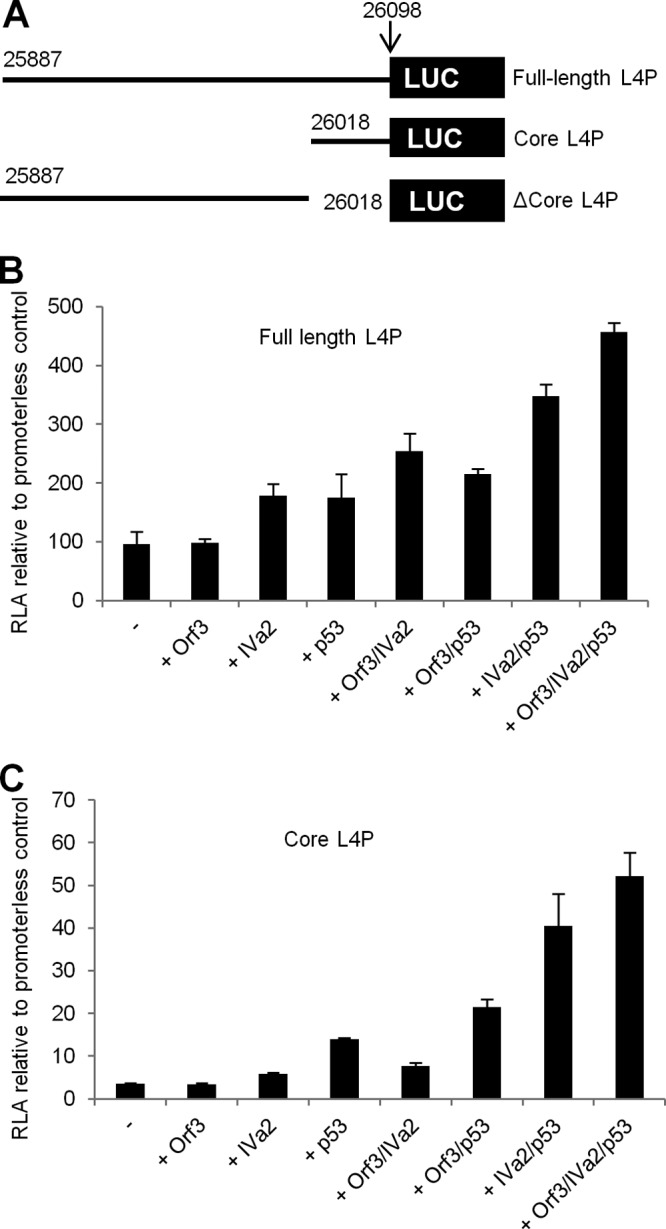
The L4 promoter is activated by p53. (A) Schematic showing the L4 promoter reporter constructs used in this study. Plasmids were constructed using L4P DNA at the indicated positions from wild-type Ad5 genome pTG-Ad3602 subcloned into the promoterless luciferase reporter plasmid pGL-3-Basic. (B) 293 cells were transfected either with pGL3-Basic or with full-length L4P alongside expression plasmids for the proteins indicated below the graph. All transfections included a standard amount of pCMV-βgal and, total DNA input was kept constant by addition of empty vector pCI-neo as required. Cells were harvested 24 h later, and reporter gene activity was assayed. Data are presented as relative luciferase activity (RLA), normalized to the pGL-3-Basic control RLA, which was set at 1. Error bars indicate the standard deviations for three replicate samples, and data are representative of a minimum of three independent repeat experiments. (C) As for panel B, except that the core L4P reporter (positions 26018 to 26098) was used.

It has previously been reported that Ad5 L4P activity is increased by transfection of fragmented DNA, indicating that this promoter is responsive to at least one cellular factor that is involved in the response to double-strand breaks (DSB) (16). One possible factor was the cellular tumor suppressor p53, a global regulator of cellular responses to stress that is known to be activated by DNA damage. The effect of p53 overexpression on the activity of the L4P was therefore investigated using luciferase reporter constructs in 293 cells (16) (Fig. 1A). Exogenous expression of p53 alone was sufficient to induce activity of both full-length (Fig. 1B) and core (Fig. 1C) L4P reporter constructs 2- to 3-fold. When added p53 was combined with exogenous expression of the previously defined L4P activators E4 Orf3 and IVa2 (12), both full-length and core L4P reporter plasmids displayed enhanced activity that was greater than when any of these factors was present alone.

**Fig 2:**
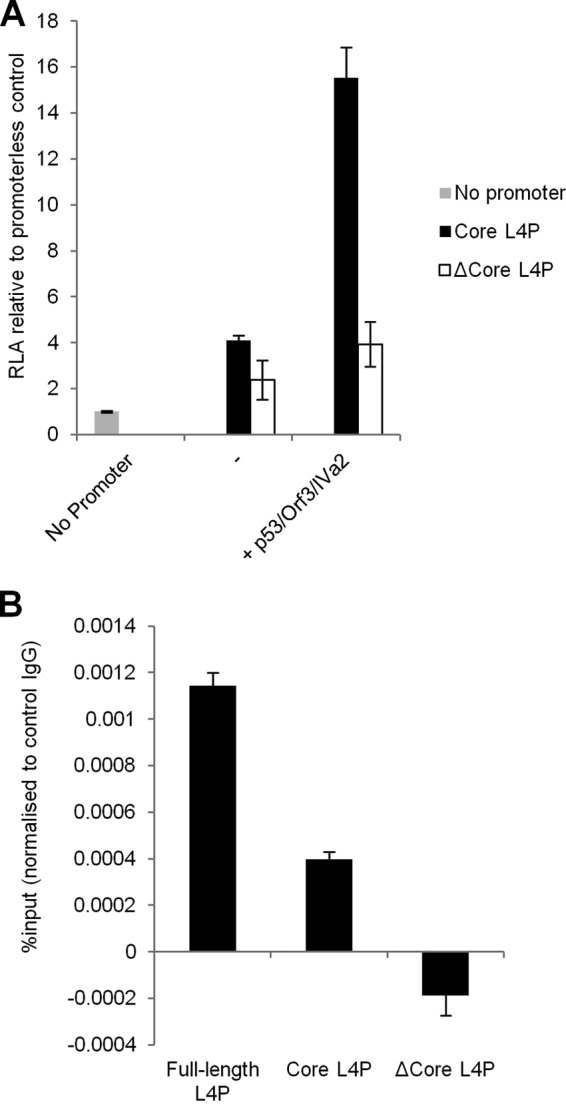
p53 binds with the core L4 promoter (positions 26018 to 26098). (A) 293 cells were transfected with pGL-3-Basic (no promoter), core L4P, or ΔCore L4P reporter plasmids alongside pCI-neo (−) or a cocktail of Orf3, IVa2, and p53 expression plasmids, and extracts were assayed for reporter gene activity. Other details are as in Fig. 1B. Error bars indicate the standard deviations for three replicate samples, and data are representative of a minimum of two experiments. (B) 293 cells were transfected with the L4P reporter plasmids indicated. Twenty-four hours later, cells were fixed and processed for ChIP analysis to determine p53 binding, expressed as the percentage of L4P DNA present in the lysate (see Materials and Methods).

We next compared the activity of the core L4P to that of a construct that contained the full-length promoter exactly lacking the core promoter sequence (ΔCore) (Fig. 1A). Basal promoter activities of the constructs were comparable (Fig. 2A). As before, coexpression of p53, E4 Orf3 and IVa2 strongly induced core promoter activity (Fig. 2A). In contrast, expression of these proteins caused only a marginal increase in activity of the ΔCore construct. These data indicate that the core L4P promoter contains the sequences required for the induction of activity observed upon overexpression of p53, E4 Orf3, and IVa2.

To investigate the possibility of p53 binding to L4P, the association of endogenous p53 with the three L4P reporter constructs was examined by chromatin immunoprecipitation (ChIP) (Fig. 2B). Quantitative PCR analysis of immunoprecipitated DNA revealed that the L4P sequence was significantly enriched in anti-p53 antibody precipitates over control antibody precipitates when either full-length or core L4P reporters were used as input. Enrichment of the core promoter was less efficient than that of the full-length L4P, possibly reflecting a requirement for additional sequences outside the core promoter for maximum efficiency of p53 binding. In contrast, no L4P sequence was immunoprecipitated when the ΔCore L4P construct was tested. Taken together, these data demonstrate that p53 is able to induce L4P activity by associating with the core region of the promoter.

### L4P activity is inhibited by depletion of endogenous p53.

**Fig 3:**
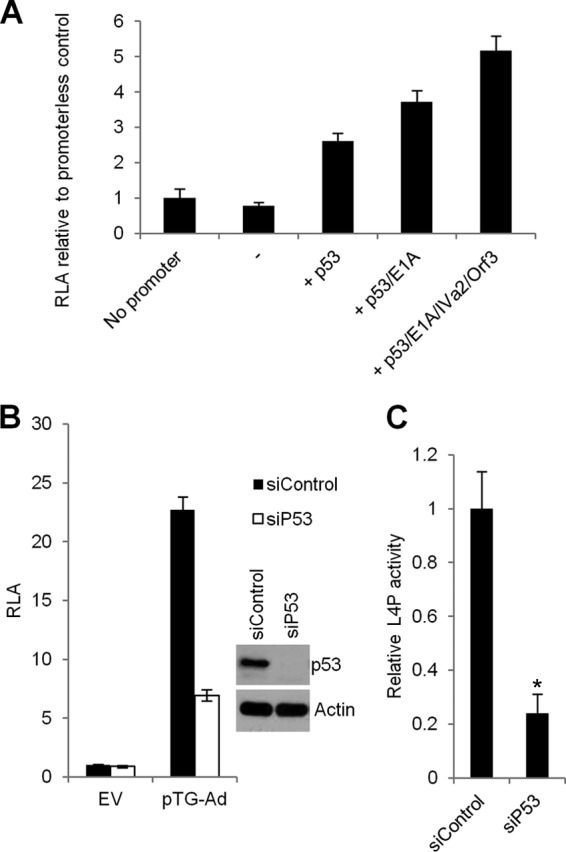
p53 can regulate the L4 promoter in U2OS cells. (A) U2OS cells were transfected with pGL-3-Basic (no promoter) or full-length L4P together with plasmids expressing the proteins indicated, and lysates were analyzed for reporter gene activity (RLA). Other details are as in Fig. 1B. Error bars indicate the standard deviations for three replicate samples. (B) U2OS cells were transfected with control siRNA or p53 siRNA. Forty-eight hours later, cells were transfected with pCI-neo empty vector (EV) or linear pTG-Ad3602. Cells were harvested after a further 24 h and lysates analyzed for reporter plasmid activity (RLA). Error bars show the standard deviations for three replicates, and data are representative of a minimum of three independent experiments. (Inset) One replicate well from the cultures used for reporter analyses was harvested for protein analysis by Western blotting with p53 and β-actin antibodies. (C) Average RLA of nine repeat readings from three independent repeat experiments whose results are shown in panel B. Data are expressed relative to the values obtained from siControl/pTG-Ad-transfected cells. Error bars indicate the standard deviations for the nine replicates, and values were subjected to Student's *t* test (2-tailed, unequal variance) *, P < 0.0001.

The reporter assays described above were carried out in 293 cells due to their documented ability to support high transfection efficiencies. However, these cells also encode a number of adenovirus proteins, including E1A and the p53-interacting protein E1B-55K, and therefore we could not discount the possibility that p53-induced activity of L4P in these cells was affected by the particular genetic background of these cells. To address this, the effect of p53 expression on full-length L4P activity was analyzed in U2OS cells, a cell line with wild-type p53 and no known endogenous adenovirus proteins. In contrast to 293 cells, basal activity of full-length L4P in U2OS cells was comparable to the promoterless control (Fig. 3A), most likely due to the absence of endogenous E1A which is a potent inducer of the L4P (16). Nonetheless, the addition of exogenous p53 was still able to increase activity of the L4P by 2- to 3-fold (Fig. 3A), consistent with the data in 293 cells (Fig. 1B). Furthermore, as in 293 cells, activity of the L4P could also be further enhanced by expression of E1A, E4 Orf3, and IVa2 alongside p53. Therefore, p53 activation of the L4P is not restricted to a single cell type and does not depend on the presence of adenovirus proteins.

We next investigated the role of endogenous p53 in the activity of the L4P. Small interfering RNAs (siRNAs) were used to deplete U2OS cells of p53 prior to transfection with linearized Ad genome and the full-length L4P reporter. Transfected Ad genomic DNA is a strong inducer of the L4P (16) and was utilized here to ensure that transfected cells produced all of the virally encoded regulators of the promoter. In cells treated with control siRNA, the addition of Ad5 genome led to a robust (>20-fold) increase in L4P activity (Fig. 3B). Upon p53 depletion, basal reporter activity was largely unaffected, but induction of the L4P by Ad5 genome was substantially inhibited. Analysis across multiple experiments yielded similar results, with p53 knockdown reducing L4P induction by Ad genome by approximately 80% with a high level of statistical significance (Fig. 3C). Therefore, endogenous p53 is a significant activator of L4P, and its depletion severely impairs the induction of the L4P by factors expressed from the Ad5 genome.

### p53 associates with the L4 promoter during Ad5 infection.

**Fig 4:**
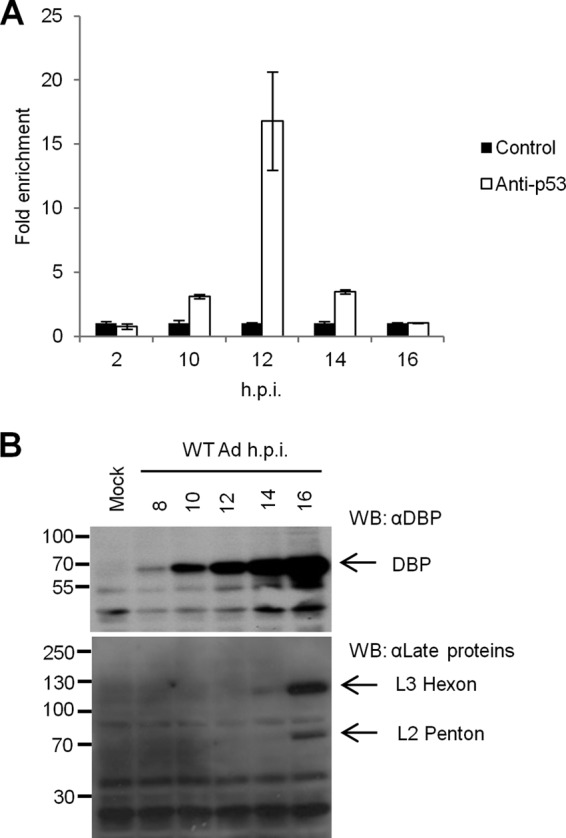
Endogenous p53 associates with the L4 promoter during Ad5 infection. (A) HeLa cells were infected at a multiplicity of infection (MOI) of 10 FFU/cell with wild-type Ad5 and then harvested at the time points indicated. Samples were then processed for ChIP analysis to determine specific p53 binding to L4P. The amount of L4P DNA recovered from anti-p53 samples is expressed relative to that detected in control antibody immunoprecipitates. Error bars indicate the standard deviations for three replicates, and the data are representative of two experiments. (B) Cells infected as described for panel A were harvested and analyzed by Western blotting for expression of Ad early and late proteins. The migration positions of protein molecular mass markers are indicated on the left, in kDa.

We next sought to detect endogenous p53 binding to adenovirus genomic DNA during productive infection by using ChIP analysis on HeLa cells infected with wild-type Ad5 (Fig. 4A). As expected, no p53 binding at the L4 promoter was detected at 2 h postinfection (hpi), a time when the L4P would be expected to be inactive. At 10 hpi, L4P DNA was enriched about 3-fold by p53 antibody over a nonspecific control. This increased to a peak at 12 hpi, when L4P was enriched >15-fold. Strikingly, p53 association with L4P was then rapidly lost, to such an extent that by 16 hpi no p53 association with the L4P was detected above background level.

A time course analysis of virus protein expression was then performed to monitor the timing of the early-late transition in Ad5 gene expression under the conditions employed here (Fig. 4B). DBP served as a representative early gene product, and accordingly this protein was present at 8 hpi and accumulated to high levels during the course of the infection. MLP activity was examined by using a polyclonal antiserum raised against the adenovirus late proteins (AdJLB1). L3 hexon was first detectable at 14 hpi, and by 16 hpi L2 penton expression was also detected. Hence, the onset of late gene expression correlated well with the period of time when p53 was associated with the L4 promoter.

### Depletion of p53 severely reduces Ad5 late gene expression.

**Fig 5:**
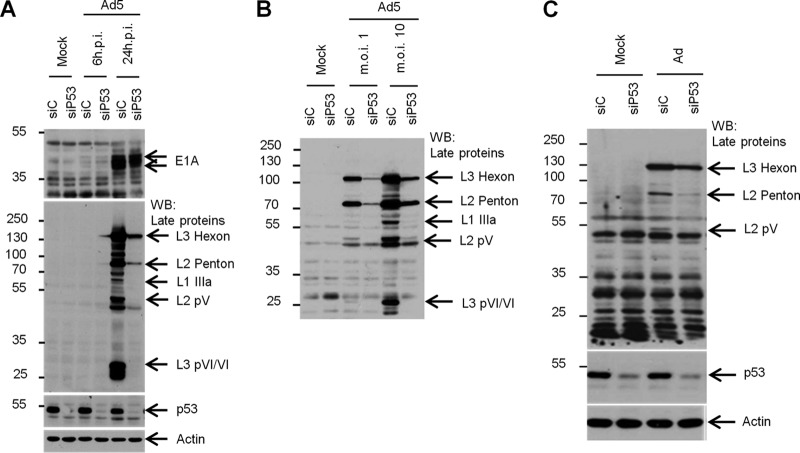
p53 is required for efficient Ad5 late gene expression. (A) HeLa cells were transfected with control or p53 siRNAs. Twenty-four hours later, cells were trypsinized and replated into 24-well plates and cultured for 24 h before mock infection or infection with wild-type Ad5 at an MOI of 10 FFU/cell. Cells were harvested at 6 and 24 hpi, and protein lysates were analyzed by SDS-PAGE and Western blotting with antibodies to E1A, late proteins, p53, or actin. For each blot, the protein(s) of interest is highlighted with arrows. (B) As for panel A, but infections were performed at a multiplicity of infection of either 1 or 10 FFU/cell and harvested at 24 hpi. (C) MRC5 cells were transfected with control or p53 siRNA. Forty-eight hours later, cells were infected with wild-type Ad5 at an MOI of 10 FFU/cell. Cells were harvested 24 hpi, and protein lysates were analyzed by SDS-PAGE and Western blotting with antibodies to late proteins, p53, or β-actin. Protein size markers are on the left of each panel, in kDa.

The data above strongly suggested that p53 plays a role in the coordinated expression of Ad5 late proteins via its regulation of L4P. To test this possibility, we examined Ad5 protein expression in HeLa cells that had been depleted of p53 by siRNA treatment prior to infection (Fig. 5A). We analyzed expression of the virus immediate early gene product E1A to gauge the progress of infection in the early phase. At both 6 and 24 hpi, at a high multiplicity of infection, levels of E1A were comparable between control and p53-depleted cells, indicating that the early phase of infection was not affected by depletion of p53. However, at 24 hpi, when the late phase was well established, significantly reduced levels of all of the viral late proteins were observed in cells depleted of p53 in comparison with the control cells (Fig. 5A). Further analysis of repeat experiments demonstrated that this defect was apparent at both low and high multiplicities of infection (Fig. 5B). Although all late protein expression was reduced upon p53 depletion, L2 pV and in particular L3 pVI were reduced to very low or undetectable levels. In control cells, L3 pVI expression was greater than that of L2 penton (Fig. 5A), yet upon p53 depletion, levels of L3 pVI were undetectable despite L2 penton expression being still clearly evident. A recently published study has suggested that, in normal cells, depletion of p53 does not affect adenovirus late protein expression (32), raising the possibility that our results might reflect an aspect of the unique biology of immortalized cell lines. To address this, we tested the effect of p53 depletion on Ad5 infection in MRC5 cells; these are a strain of normal human diploid fibroblasts from embryo lung tissue (40). Consistent with our observations in HeLa cells, depletion of p53 prior to infection led to significantly reduced levels of L3 hexon, L2 penton, and L2 pV (Fig. 5C), as was observed in immortalized cells. In summary, these data demonstrate that p53 is required for both efficient late gene expression and the correct balance of adenovirus late protein expression and that this is true in both immortalized and normal diploid cells.

### L4-22K and 33K negatively regulate L4P activity.

The transient association of p53 with L4P during Ad5 infection might be explained by two processes. First, 22K/33K-mediated stimulation of the MLP (7–9) would in turn drive transcription of the MLTU across L4P and thus lead to clearance of any bound regulatory proteins by RNA polymerase and its associated factors. Alternatively, but not mutually exclusively, L4P could be subject to a negative feedback mechanism mediated by its gene products L4-22K and L4-33K. In order to test the latter possibility, we investigated the effect of L4-22K and L4-33K expression on L4P reporter constructs.

**Fig 6:**
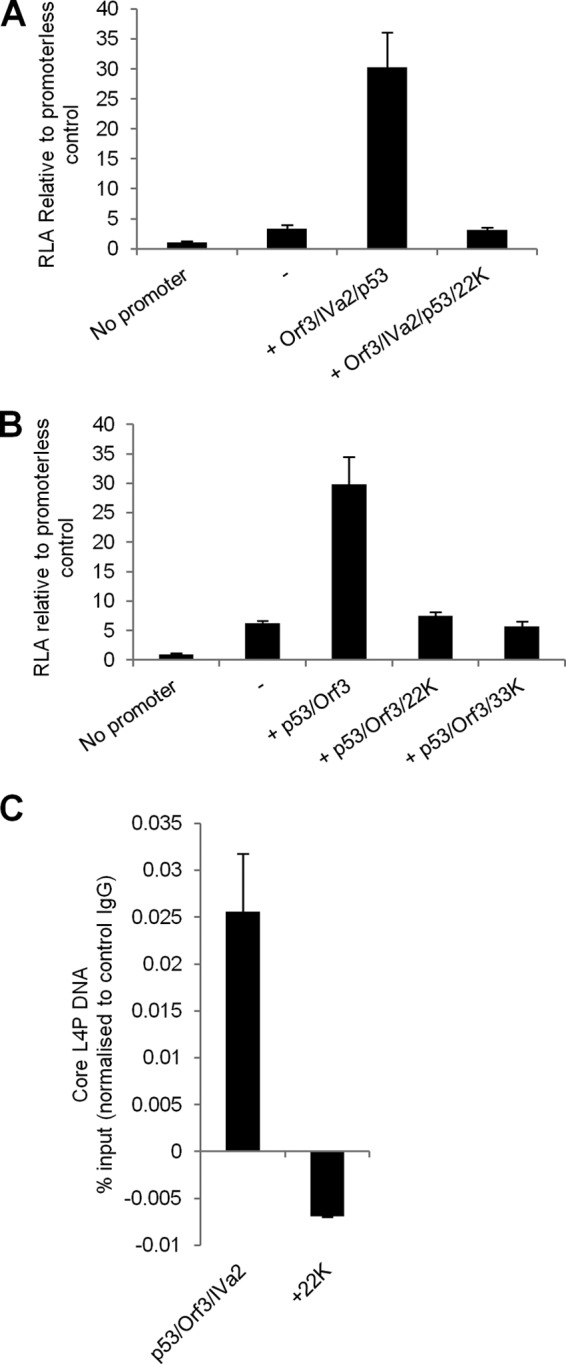
L4-22K and L4-33K inhibit L4P activity. (A and B) 293 cells were transfected with either pGL-3-Basic (no promoter) or with core L4P reporter plasmid plus plasmids expressing the proteins indicated below the graph. After 24 h, cells were assayed for reporter activity (RLA), and data were expressed relative to the activity of the promoterless control. Error bars indicate the standard deviations for three biological replicates. (C) 293 cells were transfected with full-length L4P reporter plasmid plus plasmids expressing the indicated proteins for 24 h before being harvested for ChIP analysis. Samples were subjected to ChIP analysis to determine p53 binding, expressed as the percentage of L4P DNA present in the lysate (see Materials and Methods). Error bars indicate the standard deviations for three technical replicates, and data are representative of two independent repeat experiments.

As before, coexpression of p53, E4 Orf3 and IVa2 led to a striking (10-fold) increase in the activity of the core L4P reporter in 293 cells (Fig. 6A). However, promoter activation was completely inhibited upon the additional expression of L4-22K FLAG. Given the documented interactions between IVa2 and L4-22K (7, 9, 42), it was possible that L4-22K was acting by sequestering IVa2 away from the L4P promoter sequence. In this scenario, the presence of IVa2 would be necessary for L4-22K to inhibit L4P activation. To test this, core L4P was activated by p53 and E4 Orf3 only, and the effect of L4-22K on activity was examined (Fig. 6B). While p53 in conjunction with E4 Orf3 activated core L4P approximately 5- to 6-fold, when either L4-22K FLAG or the related L4-33K FLAG protein was coexpressed with these proteins, activation was completely abrogated. Thus, IVa2 is not required for L4-22K mediated inhibition. However, L4-22K FLAG and L4-33K FLAG also inhibited the induction of the L4P by IVa2 alone (data not shown). These data suggested that no one viral factor was the target for L4-22K action.

Since the cellular activator p53 was present (either endogenous or with additional exogenous expression) in both situations where L4-22K-mediated inhibition of L4P was observed, we finally sought to determine the effect of L4-22K on p53 association with L4P, using 293 cells transfected with the full-length L4P reporter and the necessary expression plasmids (Fig. 6C). p53 binding of L4P sequence was significant when p53, IVa2, and E4 Orf3 were coexpressed. However, upon addition of L4-22K, this p53 binding to L4P was completely abolished. Thus, L4 22K directly inhibits p53 association with the L4 promoter, accounting for the transient pattern of p53 binding during Ad5 infection.

## DISCUSSION

During infection, Ad5 proceeds through a controlled and coordinated pattern of gene expression. In this study, we have demonstrated that the human tumor suppressor protein p53 contributes to this control by regulating the activity of the L4 promoter, L4P, and thus entry into the late phase of the Ad5 life cycle. It does this by binding to one or more specific target sequences within L4P. p53 activates the promoter in synergy with virus-coded activators, but importantly, p53 alone can activate L4P in the absence of any viral proteins. These findings provide a rational basis for previous reports suggesting a positive role for p53 in the adenovirus life cycle.

In fact, previous studies have provided conflicting data on the role of p53 in Ad5 infection. While several studies have shown a positive effect of p53 on late gene expression and cytopathic effect in tumor cell lines (29, 30), a recent study in two types of normal human cells concluded that Ad5 late gene expression occurs as efficiently in p53-depleted cells as in control cells (32), and in one of these cell types, virus yield was also shown to be unaffected; these authors argued that any positive role for p53 found previously was an artifact of working in tumor cell lines and/or of making comparisons between cells of differing p53 status that were in other respects not well matched. Certainly, there is no correlation between the yield of wild-type Ad5 infection and the p53 status of the cell (43), though this is unsurprising given the number of other host factors that also contribute to the intrinsic productivity of infection. Here, we found that depletion of p53 by siRNA in both immortalized and nonimmortalized cells led to significantly reduced virus late gene expression.

The apparent contradiction between these findings and the results of Chahal and Flint (32) is possibly due to differences in the methods employed; the normal cells used in the two studies were not identical, and the nature of the assays, the time points analyzed, and the viruses employed also differed. Since p53 is expressed in a number of isoforms, we considered whether the studies might differ as to which isoforms were targeted for knockdown, but although the p53-specific siRNA/short hairpin RNA (shRNA) sequences used do differ, their sequences overlap, and both should target all isoforms. In fact, the findings of Chahal and Flint and those reported here can be reconciled on the basis that in healthy dividing cells, the requirement for L4P activity, and hence for p53 activation, is only transient, and once the MLP is activated by L4 proteins acting with IVa2, late gene expression would be expected to gradually recover from a poor start due to the lack of p53 activation of L4P.

When Royds et al. found that the presence of p53 led to enhanced viral late gene expression (30), they concluded that this was due to p53 cooperating with E1A to upregulate MLP activity. Our data, however, indicate that the primary effect of p53 on late gene expression may occur earlier than this, at activation of the L4P, rather than the MLP. In fact, the data of Royds et al. are not inconsistent with those presented here, as it is entirely possible that p53 may regulate both L4P and MLP; indeed, two other known activators of L4P, E1A and IVa2, also activate MLP. Unlike the MLP however, where E1A is necessary for p53-mediated activation, overexpression of p53 alone was sufficient to induce L4P activity, indicating that the methods of activation of the two promoters by p53 are different.

The fact that depletion of p53 prior to infection led to significantly reduced levels of virus late gene expression in both HeLa and MRC5 cells does not directly indicate whether the effect of p53 is at the level of MLP or L4P activation. However, we also found that the expression of some late gene products, particularly L2 pV and L3 pVI, was disproportionately reduced upon p53 depletion in comparison to the L3 hexon and L2 penton. Studies using L4-22K- and L4-33K-deficient viruses have demonstrated that the expression of both L2 pV and L3 pVI is dependent on the expression of L4-22K and L4-33K (13–15) and that an L4P mutant genome, late protein expression from which can be partially restored upon complementation in *trans* with L4-22K, requires further addition of L4-33K for expression of L3 pVI (16). The lack of L2 pV and L3 pVI expression during p53 depletion is therefore consistent with L4-22K and L4-33K being absent or expressed at low levels, as would be expected if L4P activity was impaired. The reduction in L3 hexon and L2 penton levels can in turn be explained by MLP activity being decreased due to the absence of L4-22K and L4-33K to act as activators (8, 9), though a direct contribution from the loss of p53 acting at the MLP in concert with E1A cannot be excluded (30).

Our ChIP analyses demonstrate that p53 association with L4P peaks at 12 hpi before being rapidly lost, a period just prior to the detected onset of full late gene expression, when L4P would be expected to be active. p53 is present in the cell throughout the early phase of infection, so it is interesting to consider how p53 is recruited to the L4P specifically at this time during infection. Levels of p53 initially increase following infection as a result of the activity of E1A proteins (18, 19), but p53's transcription activation function is rapidly blocked due to its binding by E1B-55K (22). However, this inhibition of p53 is temporarily relieved by E4 Orf3 before the slightly later expression of E4 Orf6 allows formation of the E1B-55K/E4 Orf6 ubiquitin ligase complex that triggers p53 degradation (33). This temporal cascade of interactions with p53 provides a potential mechanism whereby p53 could be liberated for binding to the L4P at the intermediate stage in infection. In infected HeLa cells, E4 Orf3 was detectable from 9 hpi, whereas E4 Orf6 was detectable only from 15 hpi (33). The timing during infection of p53 association with the L4P observed here therefore coincides with the period when its activity would be transiently released from E1B-55K-mediated inhibition. As well as releasing p53 from E1B-55K, E4 Orf3 may also promote the association of p53 with L4P over its binding to cellular chromatin due to E4 Orf3's ability to induce heterochromatin formation at p53-dependent promoters and thus inhibit p53 activation (25). Since E4 Orf3 has no documented ability to bind DNA, its ability to influence p53 function provides an attractive mechanism whereby its activation of L4P may be explained. Further studies will be required to establish whether this is the case.

The precise sequence elements required for the association of p53 with the L4P core promoter remain to be determined. *In silico* prediction of cellular transcription factor binding sites indicates that there are at least two potential p53-binding sites within the core promoter (data not shown). Further studies will be required to determine if these sites are indeed the target of p53 during infection.

The association of p53 with the L4P is rapidly lost during the course of infection. This may be due to two reasons that are not mutually exclusive. First, the timing of the loss of p53 from L4P broadly corresponds with the expected onset of E4 Orf6 expression (33). It is therefore possible that p53 degradation by the action of E1B-55K/E4 Orf6 complex contributes to the loss of p53 from the L4P subsequent to its activation. However, Grand and colleagues observed that in A549 cells infected at a multiplicity of infection greater than that used in this study, significant amounts of p53 remained at 18 hpi (44), and in our study, significant amounts of p53 remained detectable at 24 hpi in both HeLa and MRC5 cells (Fig. 5), suggesting that loss of p53 from L4P by 16 hpi is not due to its degradation. Second, our results showed that L4-22K and L4-33K were both capable of inhibiting L4P activity and expression of L4-22K was sufficient to abolish the association of p53 with the L4P. Thus, the transient nature of p53 binding to L4P may be due primarily to L4P expression products acting in a negative feedback loop. L4-22K and/or L4-33K have been shown previously to interact with IVa2 in a complex termed DEF-A that can bind sequences within the major late promoter (8, 9). Given that IVa2 is an activator of L4P, this raised the possibility that L4-22K inhibits L4P via its interaction with IVa2. However, the presence of IVa2 in the cocktail of L4P inducers was not required for inhibition of L4P by L4-22K and L4-33K, as activity was severely abrogated by these proteins even in assays where IVa2 was absent, demonstrating that repression of L4P is not dependent on the sequestration of IVa2.

We propose a model whereby cellular p53 is utilized by adenovirus to optimize the timing of late gene expression. p53 acts in conjunction with E1A, E4 Orf3, and IVa2 to activate L4P to drive expression of L4-22K and L4-33K before it is itself degraded by the E4 Orf6/E1B-55K complex. L4-22K and L4-33K in turn act to limit expression from the L4P by preventing p53 binding, while at the same time contributing to maximal MLTU expression from the MLP (8, 9). Further studies of this mechanism are focusing on the sequence elements required for regulation and the identification of any further regulatory proteins, in order to understand the role of L4P in the biology of adenoviruses more fully.
